# SpatialQC: automated quality control for spatial transcriptome data

**DOI:** 10.1093/bioinformatics/btae458

**Published:** 2024-07-25

**Authors:** Guangyao Mao, Yi Yang, Zhuojuan Luo, Chengqi Lin, Peng Xie

**Affiliations:** Key Laboratory of Developmental Genes and Human Disease, School of Life Science and Technology, Southeast University, Nanjing 210000, China; Co-innovation Center of Neuroregeneration, Nantong University, Nantong 226000, China; Key Laboratory of Developmental Genes and Human Disease, School of Life Science and Technology, Southeast University, Nanjing 210000, China; Key Laboratory of Developmental Genes and Human Disease, School of Life Science and Technology, Southeast University, Nanjing 210000, China; Co-innovation Center of Neuroregeneration, Nantong University, Nantong 226000, China; Center of Reproductive Medicine, Fujian Maternity and Child Health Hospital, Fuzhou 350000, China; Shenzhen Research Institute, Southeast University, Shenzhen, China; Key Laboratory of Developmental Genes and Human Disease, School of Life Science and Technology, Southeast University, Nanjing 210000, China; Co-innovation Center of Neuroregeneration, Nantong University, Nantong 226000, China; Center of Reproductive Medicine, Fujian Maternity and Child Health Hospital, Fuzhou 350000, China; Shenzhen Research Institute, Southeast University, Shenzhen, China; School of Biological Science & Medical Engineering, Southeast University, Nanjing 518000, China

## Abstract

**Summary:**

The advent of spatial transcriptomics has revolutionized our understanding of the spatial heterogeneity in tissues, providing unprecedented insights into the cellular and molecular mechanisms underlying biological processes. Although quality control (QC) critical for downstream data analyses, there is currently a lack of specialized tools for one-stop spatial transcriptome QC. Here, we introduce SpatialQC, a one-stop QC pipeline, which generates comprehensive QC reports and produces clean data in an interactive fashion. SpatialQC is widely applicable to spatial transcriptomic techniques.

**Availability and implementation:**

source code and user manuals are available via https://github.com/mgy520/spatialQC, and deposited on Zenodo (https://doi.org/10.5281/zenodo.12634669).

## 1 Introduction

Spatial transcriptomics is a crucial and expanding field and a crucial technique in contemporary biological and biomedical research. It offers unparalleled insights into our understanding of complex biological processes and disease mechanisms (Williams *et al.* 2022, Tian *et al.* 2023). The rising of spatial transcriptome techniques, such as stereo-seq (Chen *et al.* 2022), enables the investigation of the spatial gene expression distribution at subcellular resolution with large visual field. However, these techniques suffer from major drawbacks, especially the compromised sequencing depth of single cells and uneven sequencing quality over large samples (Moses and Pachter 2022). Thus, systematic quality evaluation and data cleaning is the first step toward reliable analysis of spatial transcriptomic data. Before in-depth investigation, researchers need to understand the quality distribution (including sequencing depth, library complexity, etc.) over their dataset and to identify their data of interest. To our knowledge, the field lacks in computational tools which serve the above-mentioned purposes.

Quality control is paramount for scRNA-seq and spatial transcriptomics, as accurate detection and removal of technical artifacts form the foundation for downstream analysis. While several QC tools such as scQCEA (Nassiri *et al.* 2023) and popsicleR (Grandi *et al.* 2022) have been developed for scRNA-seq data primarily from the 10X Genomics Cell Ranger pipeline, there is currently a gap in QC software tailored for spatial transcriptomics: (i) abnormal spatial distribution of data quality, which existing methods fail to detect; (ii) assessment of quality at the level of tissue sections, which existing methods cannot accomplish; (iii) variance in sequencing depth across different spatial transcriptomics platforms, requiring quantifiable standards for parameterization, which is more pronounced than for single-cell data. Therefore, there is an urgent need for dedicated QC methods tailored specifically for spatial transcriptomics data.

Here, we introduce SpatialQC, a quality control software designed for assisting researchers in rapidly and accurately evaluating the quality of their spatial transcriptomics data. SpatialQC provides a one-click solution for automating quality assessment, data cleaning, and report generation. SpatialQC is designed to solve the specific needs of the spatial transcriptomic data, as mentioned above. It calculates a series of quality metrics, the spatial distribution of which can be inspected, in the QC report, for spatial anomaly detection. It performs quality comparison between tissue sections, allowing for efficient identification of questionable slices. It provides a set of adjustable parameters and comprehensive tests to facilitate informed parameterization. Experiments demonstrate that SpatialQC significantly improves data quality across major spatial transcriptomic techniques. In addition, SpatialQC supports various widely used input data formats, including “anndata” (Wolf *et al.* 2018, Palla *et al.* 2022), “Seurat” (Stuart *et al.* 2019), “SingleCellExperiment” (Amezquita *et al.* 2020), “SpatialExperiment” (Righelli *et al.* 2022), and “gem” (Gong *et al.* 2024).

## 2 Materials and methods

### 2.1 Overall structure of SpatialQC

SpatialQC follows a systematic workflow comprising three main modules: cell/spot scoring, data filtering, and report generation.

#### 2.1.1 Cell/spot scoring module

The cell/spot scoring module in SpatialQC evaluates the quality of individual cells/spots through predefined criteria. Metrics such as mitochondrial gene percentage, gene counts, total RNA counts, and marker gene detection ratios are utilized for this assessment. In addition, a doublet score is calculated by Scrublet (Wolock *et al.* 2019) to identify potential doublets within the dataset (Supplementary Table S1).

#### 2.1.2 Data filtering module

**Figure 1.**
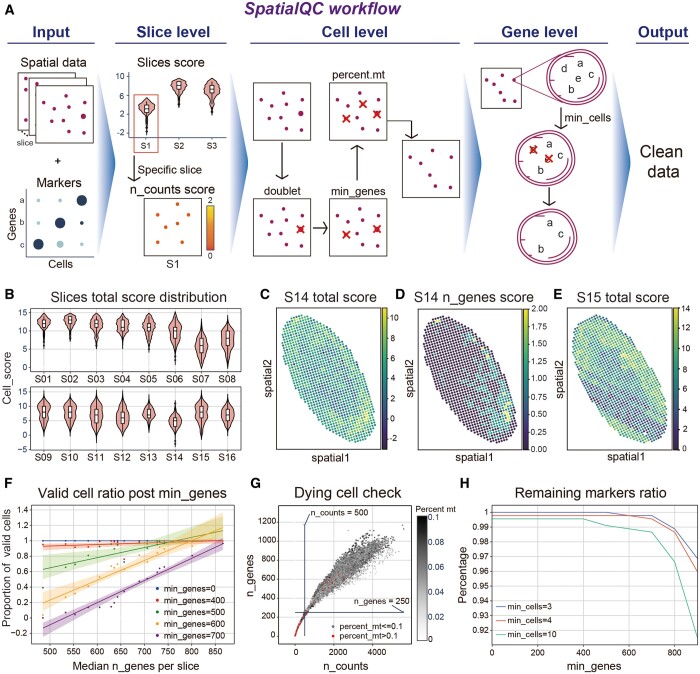
Workflow and examples of SpatialQC: (A) SpatialQC workflow contains input, filtered logic, and output. (B) Violin plot of the distribution of slice scores. (C) Spatial distribution of the total score for s14. (D) Spatial distribution of the n_genes score for s14. (E) Spatial distribution of the total score for s15. (F) The proportion of valid cells per slice, filtered by the corresponding min_genes. (G) The scatter plot of n_counts versus n_genes in each cell. (H) The line plot of the proportion of markers versus min_genes on the different min_cells.

SpatialQC performs data cleaning with three sequential steps: slice-, cell- and gene-level filtering (Fig. 1A). With user-defined parame-ters: min_score, min_genes, and min_cells (described below), the output of SpatialQC is a valid dataset in “anndata” format.

**Step 1**: Slice-level filtering. For spatial transcriptomic data, especially 3D data, samples are separately sliced and sequenced, leading to variable slice-wise data quality. SpatialQC identifies and removes invalid slices. It scores cell/spot in a slice and filter out the slice, if the median score is <5 (threshold adjustable). All the single cells of valid slices are kept for further steps.

**Step 2**: Cell-level filtering. SpatialQC filters the remaining cells using parameter min_genes. We have preset different thresholds tailored to different platforms to optimize performance. For example, to our experience, we recommend setting the value of min_genes so that >70% cells, of most valid slices, remain for Stereo-seq (Fig. 1F). In addition, SpatialQC removes cells identified as doublets and those with mitochondrial ratios exceeding 10% (threshold adjustable).

**Step 3**: Gene-level filtering. SpatialQC select genes detected in more than a minimum number of cells (min_cells). With the pre-defined marker gene set (See Section 2.2 for details), we recommend choosing the value of min_cells so that >99% (threshold adjustable) of marker genes remain.

#### 2.1.3 Report generation module

This module leverages various libraries and technologies to generate visually appealing and informative reports. The body of the report contains 14 buttons (Supplementary Table S2), a slice drop-down selection box, and an image display interface. The content of the report can be summarized in three parts.

**Data summary:** The HTML report begins with a summary of the input dataset, providing key statistics such as the total number of cells/spots, genes detected, and other relevant metadata.

**Quality assessment visualizations:** SpatialQC generates interactive visualizations to illustrate the quality assessment metrics used during the cell/spot scoring phase. These visualizations may include histograms, scatter plots, and box plots depicting metrics such as gene counts, mitochondrial ratios, and marker gene detection proportions.

**Parameter assessment:** The report provides a comprehensive analysis of the impact of different parameter combinations on the dataset. It includes insights such as the number of valid cells/spots retained after filtering, the proportion of valid cells/spots preserved in each slice, and the percentage of marker genes retained post-filtering. These detailed analyses empower users to make informed decisions when selecting parameters, enabling them to tailor the filtering criteria to their specific dataset characteristics.

### 2.2 Marker gene set

Several steps of SpatialQC requires a pre-defined marker gene set, which can be provided by the user. The use may choose to use marker genes of the “cellmarker” database (Hu *et al.* 2022) through option “—species,—tissue_class,—tissue_type.” Alternatively, the user simply sets—markers False to disable marker genes, but in this case the “—min_cells” parameter must be specified.

### 2.3 Platform compatibility

We introduced a “—platform” parameter to SpatialQC, allowing users to specify the specific platform used in their experiment. We customize different parameter combinations for each platform based on their characteristics and assess their effectiveness on various datasets (Supplementary Figs S3 and S4). Evaluation is conducted from two perspectives: (i) the distribution of cell scores before and after filtering, where the cell score represents the sum of scores in Supplementary Table S1, and (ii) the label transfer scores before and after filtering. Label transfer is accomplished using Seurat, with each cell assigned the maximum predicted score. Score comparisons are performed using SciPy's ttest_ind (Virtanen *et al.* 2020).

### 2.4 Implementation of SpatialQC

SpatialQC is a software built in Python. It uses Scanpy to process spatial transcriptome data and plotly for plotting. SpatialQC uses the Joblib module to speed up processing. SpatialQC supports multiple input formats of generated by Python- or R-based packages, and converts them to “anndata” using “anndata2ri” (Wolf *et al.* 2018). The output of SpatialQC contains filtered data and HTML interactive reports. The HTML is based on CSS and JavaScript, and the interactive charts are rendered by Plotly.js.

### 2.5 Sample data and preprocessing

The stereo-seq data used to generate Fig. 1 comes from the StomicsDB database (Xu *et al.* 2023), and the query number is STDS0000060. The sequencing tissue was Drosophila E14-16h embryos, and the sequencing platform was DNBSEQ-T1. The example Stereo-seq data consists of 16 slices containing 15 295 cells and 13 668 genes. The corresponding 451 marker genes are derived from the differentially expressed genes identified through scRNA-seq of Drosophila E14-16h embryos (Calderon *et al.* 2022). The raw stereo sequence data and 451 markers were used as sample input to SpatialQC to generate Fig. 1.

## 3 Results

We performed SpatialQC on stereo-seq data from the Drosophila embryo E14-E16h (Wang *et al.* 2022), with marker genes derived from scRNA-seq data of the Drosophila embryo atlas E14-E16h (Calderon *et al.* 2022). We present the results in three ways, as in SpatialQC workflow (Fig. 1A). Regarding slices, the median cell scores of most slices are above 5, while slice S14 shows the lowest quality (Fig. 1B). Upon closer inspection of the individual slice cell scores, we discovered that the low score of S14 was due to its significantly low sequencing depth (Fig. 1C and D). In addition, we observed that the low scores of S15 exhibited a localized pattern. (Fig. 1E), which suggested that there may be a potential issue. On the cellular level. Although the data depth increases with the increasement of parameter “min_genes,” the proportion of valid cells in each slice decreases (Fig. 1F), which may lead to sparsity of cells in the transcriptome. To balance data depth and number of cells, we preserve over 70% of cells in each slice. Therefore, we set the parameter min_genes to 490 here. Furthermore, we can remove dying cells based on their mitochondrial percentage (Fig. 1G). Finally, when filtering genes, setting the parameter min_cells to 3 can help prevent the loss of marker genes (Fig. 1H).

In addition, we performed SpatialQC on the slide-seq raw data (Supplementary documents) for the E8.5 mouse embryos (Sampath Kumar *et al.* 2023). As shown in density plot (Figure S1A, B), we observed double peaks in the kernel density plots of both detected number of genes and UMI counts per cell. As the violin plot (Supplementary Fig. S1C and D) showed, there are a lot of low depth cells in this dataset. Furthermore, taking an investigation into the scores of the sagittal 07 and 28, we found that high score cells clustered together. This is because the embryo occupied only a portion of the slide-seq bead array, but most of the slide-seq bead array occupied by cells with gene counts <200 in fact is the nontissue regions (Supplementary Fig. S1E and F). Subsequently, to remove this nontissue regions, we filtered out cells with min_genes <200, adopted by Sampath Kumar in the publication (Sampath Kumar *et al.* 2023). Upon re-running SpatialQC, the double peaks in the kernel density plots of detected number of genes and UMI counts per cell disappeared (Supplementary Fig. S2A and B). Finally, using SpatialQC with the default filter parameters, we (i) retained all slices from the mouse E8.5 embryo (Supplementary Fig. S2C), (ii) filtered out cells with detected number of genes <340 (Supplementary Fig. S2D), (iii) removed cells with the mitochondrial gene ratio greater than 0.2 (Supplementary Fig. S2E), and (iv) kept genes expressed in at least 15 cells to preserve all marker genes (Supplementary Fig. S2F).

More demonstrations of SpatialQC on sequencing-based and imaging-based platforms can be found on the online tutorial. After filtration by SpatialQC, the cell quality scores (Supplementary Fig. S3) and label transfer scores (Supplementary Fig. S4) were significantly improved, across all platforms.

## 4 Conclusion

In summary, SpatialQC is a user-friendly tool for quickly assessing the quality of spatial transcriptomic data and generating clean data simultaneously. The generated QC report is presented in HTML format, featuring interactive charts, summary statistics, and detailed information for each slice. Furthermore, the QC report allows for the identification of potential issues with the data prior to conducting additional sequencing and data analysis.

## Supplementary Material

btae458_Supplementary_Data

## Data Availability

The stereo-seq data for Drosophila embryonic stages E14-E16h were obtained from StomicsDB, with the dataset identifier STDS0000060. Drosophila embryonic stages E14-E16h scRNA-seq data is available via https://shendure-web.gs.washington.edu/content/members/DEAP_website/public/.
