# Supplementary material for: SpatialQC: automated quality control for spatial transcriptome data
Source: Bioinformatics. 2024 Jul 25;40(8):btae458. doi: 10.1093/bioinformatics/btae458 (PMC11333854; doi:10.1093/bioinformatics/btae458)
Supplement: btae458_Supplementary_Data [file btae458_supplementary_data.pdf]

Supplementary Materials

Mouse E8.5 embryo slide-seq

Raw data is available at GSE197353. The code for getting raw ‘anndata’ is available at GitHub. The corresponding marker genes are derived from the differentially expressed genes identified through scRNA-Seq of mouse E8.5 embryos (Pijuan-Sala et al., 2019).

Supplementary Tables

Table S1. Cell scoring rules

Criteria	Explanation	Scoring rule
percent.mt	Mitochondrial ratio	>10%: -1<br><=10%: 0
n_genes	Number of gene types detected	Top 20%: 0<br>20%-50%: 1<br>>50%: 2
n_counts	Number of UMI counts detected	Top 20%: 0<br>20%-50%: 1<br>>50%: 2
log10GenesPerUMI	Log10(n_genes/n_counts)	<0.8: 0<br>>0.8: 1
markerDetectionRatio	Percentage of markers detected	Top 20%: 0<br>20%-50%: 1<br>50%-80%: 2<br>>80%: 3
markerProportion	The ratio of detected markers to n_genes	Top 20%: 0<br>20%-50%: 1<br>50%-80%: 2<br>>80%: 3
markerCountsRatio	The ratio of detected marker counts to n_counts	Top 20%: 0<br>20%-50%: 1<br>50%-80%: 2<br>>80%: 3
doublet	An artificial library produced by two cells instead of one	Yes: -4<br>No: 0

The top percentage in the Score column is in ascending order

Table S2. Interpretation of buttons in HTML interactive reports

Button name	Contents
Basic Statistics	Statistical ‘anndata’ slice number, cell number, number of genes, doublet cell number and marker genes related statistics
Slice Score	Box plots of each slice score.<br>Scatter plots of 8 scoring measurements per slice
n_counts Per Cell	The kernel density estimation (KDE) and box plot distribution of the n_counts of cell
n_genes Per Cell	The kernel density estimation (KDE) and box plot distribution of the n_genes of cell.
log10GenesPerUMI	The kernel density estimation (KDE) and box plot distribution of the log10GenesPerUMI of cell.
Mitochondrial	Mitochondrial ratio distribution
Mitochondrial Scatterplot	Scatter plot of relationship between n_genes~n_counts and

	mitochondrial ratio
Ribosomal	Ribosomal ratio distribution
Hemoglobin	Hemoglobin ratio distribution
Marker Proportion	Calculate the proportion of marker genes detected in each interval based on the size of n_genes.
Cell Number Post Filter	Cell number vs. min_genes trend chart.
Valid Cell Post min_genes	Proportion of valid cells per slice vs. min_genes trend chart. If too few slices, display only bar chart.
Markers Proportion Post Filter	Trend chart of marker gene proportion in 'anndata' as it varies with min_cells.
Markers Detected Post Filter	Trend chart of the remaining proportion of user-provided marker genes as min_cells varies.

Button content changes with selected slices.

Supplementary Figures

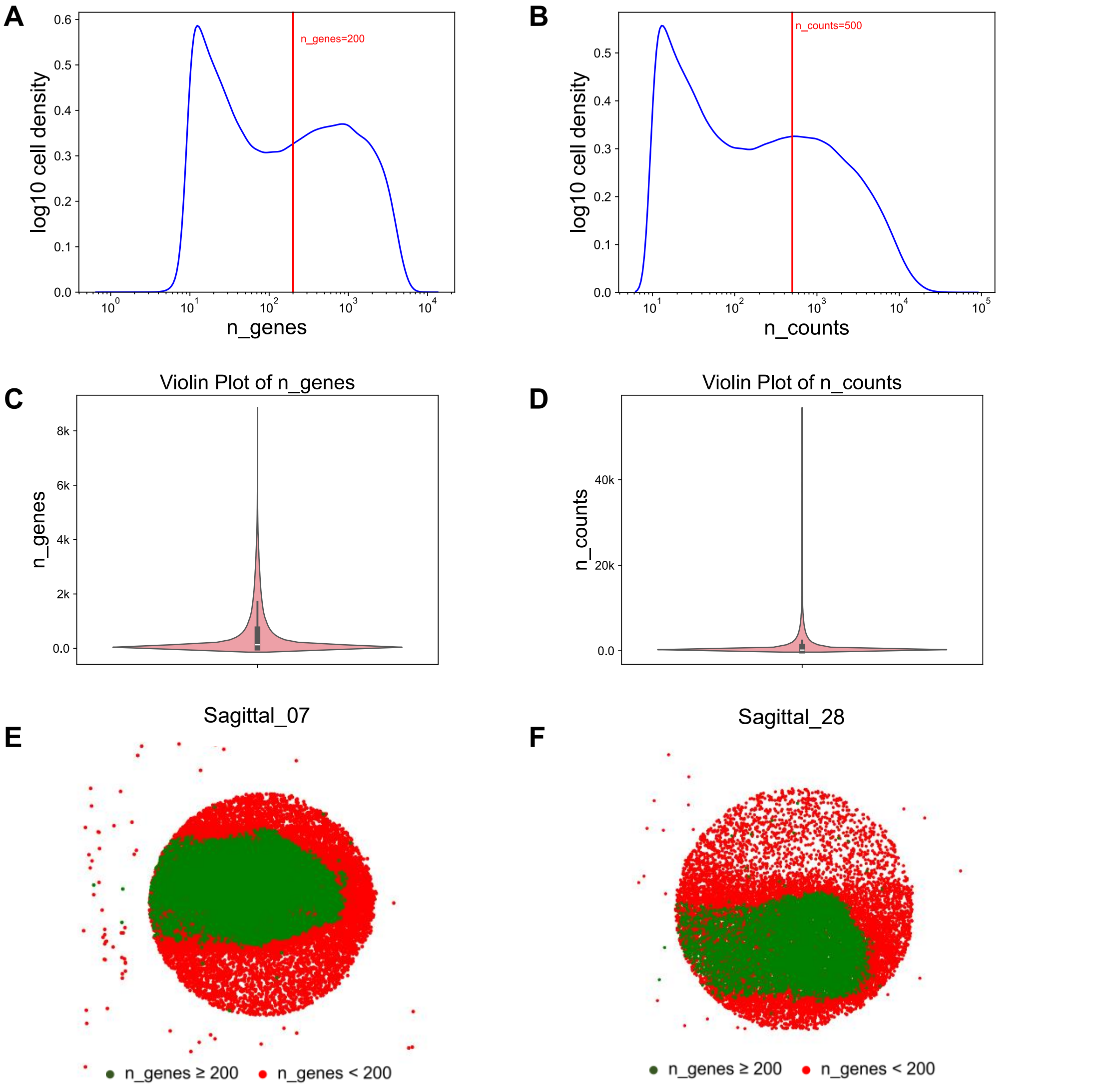

Figure S1. Quality control analysis of mouse E8.5 embryo raw data: The kernel density estimate plot

patterns of n_genes (number of detected genes in a single-cell) and n_counts (total count of RNA molecules detected in a single-cell) showed double peaks (A, B).Many cells have illegally low n_genes and n_counts (C, D). We can observe that the areas of low depth are mainly non-embryonic tissue areas (E, F).

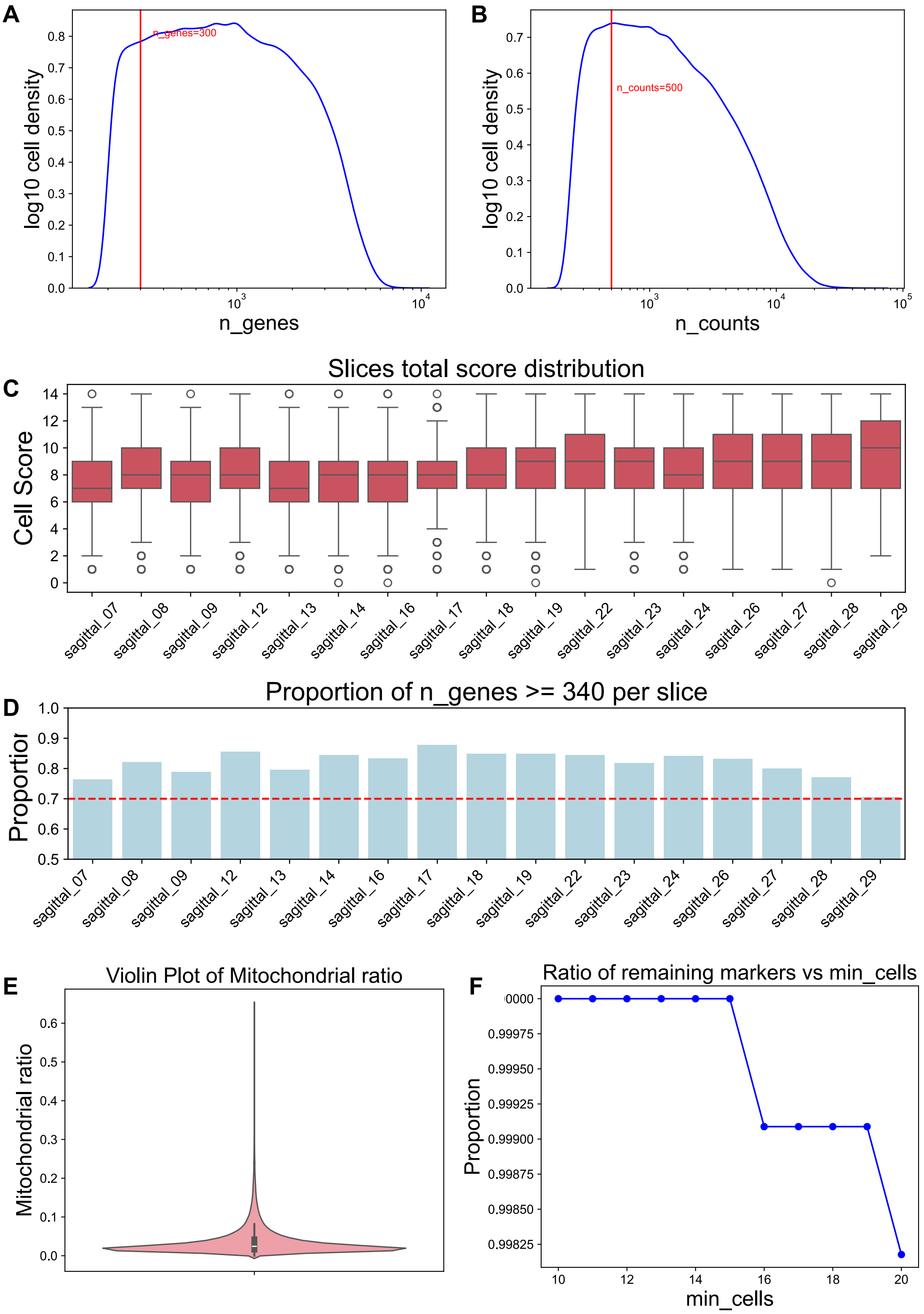

Figure S2. Quality control analysis of mouse E8.5 embryo data after initial filtration with min_genes=200: After filtering out most of the non-embryonic tissue cells, the kde patterns of n_genes

and n_counts changed to only one peak (A, B). Run SpatialQC on this data again, all slices have a median score greater than 5, and all slices are preserved (C). Setting min_genes=340 makes the percentage of active cells in all slices greater than 70% (D). We then filtered out cells with a mitochondrial gene ratio greater than 0.2 (E). Finally, min_cells=15 is set to retain all marker genes detected (F).

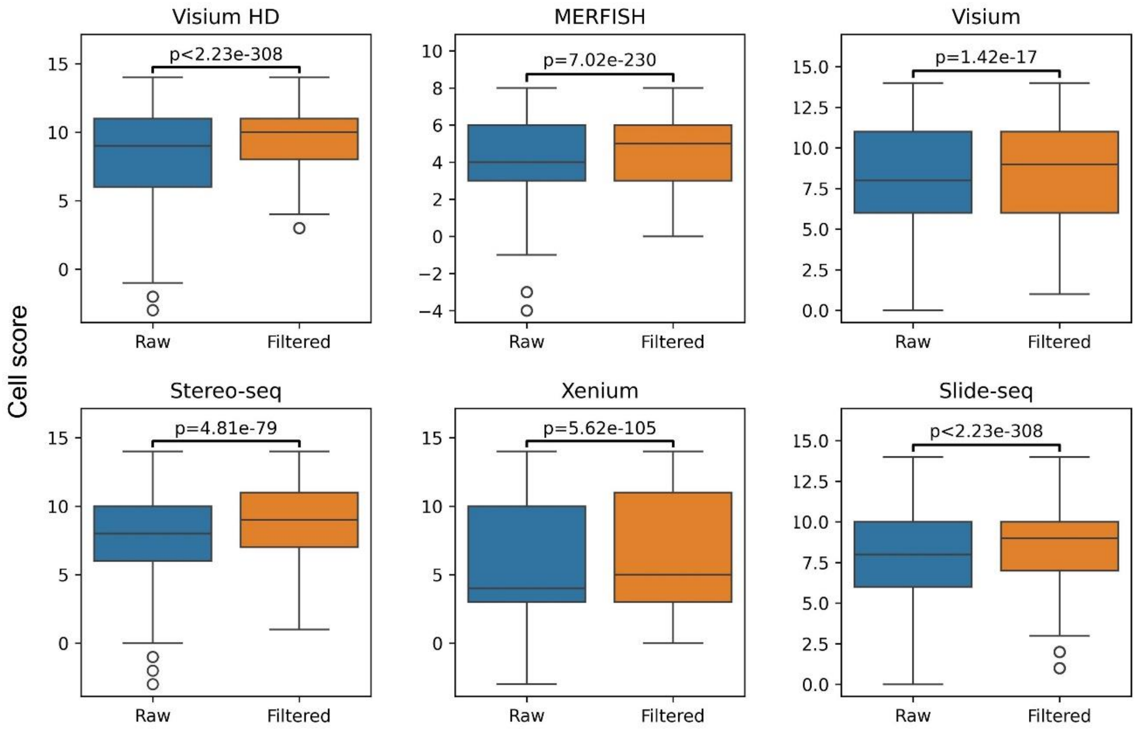

Figure S3. Comparison of cell scores before and after filtering, with p-values calculated using SciPy ttest_ind function.

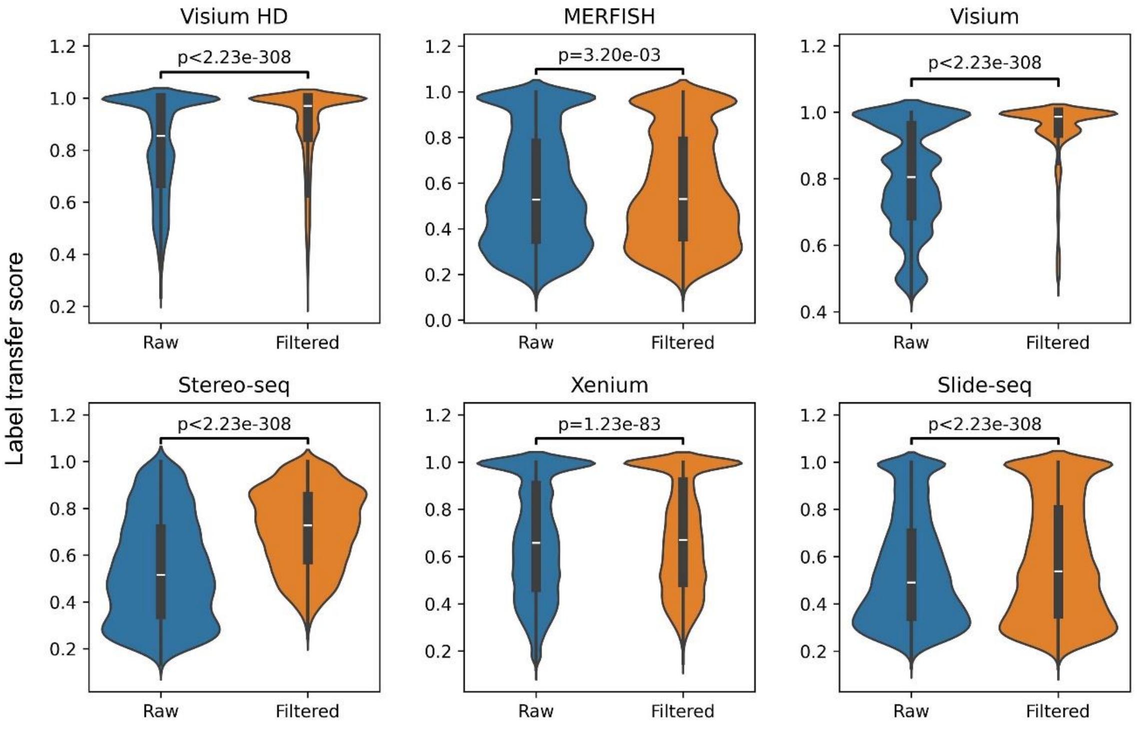

Figure S4. Comparison of label transfer score before and after filtering, with p-values calculated using SciPy ttest_ind function.

References

Pijuan-Sala,B. et al. (2019) A single-cell molecular map of mouse gastrulation and early organogenesis. Nature, 566, 490–495.
